# Surface Hydrophilicity of Poly(l-Lactide) Acid Polymer Film Changes the Human Adult Adipose Stem Cell Architecture

**DOI:** 10.3390/polym10020140

**Published:** 2018-02-01

**Authors:** Chiara Argentati, Francesco Morena, Pia Montanucci, Marco Rallini, Giuseppe Basta, Nicolino Calabrese, Riccardo Calafiore, Marino Cordellini, Carla Emiliani, Ilaria Armentano, Sabata Martino

**Affiliations:** 1 Department of Chemistry, Biology and Biotechnologies, Biochemistry and Molecular Biology Unit, University of Perugia, Via del Giochetto, 06126 Perugia, Italy; chiara.argentati89@gmail.com (C.A.); effemorena@gmail.com (F.M.); carla.emiliani@unipg.it (C.E.); 2 Section of Cardiovascular, Endocrine and Metabolic Clinical Physiology, Laboratory for Endocrine Cell Transplants and Biohybrid Organs, Department of Medicine, University of Perugia, 06126 Perugia, Italy; piamontanucci@hotmail.com (P.M.); gius.basta@gmail.com (G.B.); riccardo.calafiore@unipg.it (R.C.); 3 Civil and Environmental Engineering Department, UdR INSTM, University of Perugia, 05100 Terni, Italy; marcorallini@gmail.com; 4 Private Dental Practice, 06134 Perugia, Italy; nicperiodoc@hotmail.com; 5 Plastic and Reconstructive Surgery Unit, 06024 ASL 1 Umbria, Italy; marinocordellini@yahoo.it; 6 Department of Ecological and Biological Sciences, Tuscia University, 01100 Viterbo, Italy; ilaria.armentano@unitus.it

**Keywords:** cytoskeleton architecture, stem cell fate, regenerative medicine

## Abstract

Current knowledge indicates that the molecular cross-talk between stem cells and biomaterials guides the stem cells’ fate within a tissue engineering system. In this work, we have explored the effects of the interaction between the poly(l-lactide) acid (PLLA) polymer film and human adult adipose stem cells (hASCs), focusing on the events correlating the materials’ surface characteristics and the cells’ plasma membrane. hASCs were seeded on films of pristine PLLA polymer and on a PLLA surface modified by the radiofrequency plasma method under oxygen flow (PLLA+O_2_). Comparative experiments were performed using human bone-marrow mesenchymal stem cells (hBM-MSCs) and human umbilical matrix stem cells (hUCMSCs). After treatment with oxygen-plasma, the surface of PLLA films became hydrophilic, whereas the bulk properties were not affected. hASCs cultured on pristine PLLA polymer films acquired a spheroid conformation. On the contrary, hASCs seeded on PLLA+O_2_ film surface maintained the fibroblast-like morphology typically observed on tissue culture polystyrene. This suggests that the surface hydrophilicity is involved in the acquisition of the spheroid conformation. Noteworthy, the oxygen treatment had no effects on hBM-MSC and hUCMSC cultures and both stem cells maintained the same shape observed on PLLA films. This different behavior suggests that the biomaterial-interaction is stem cell specific.

## 1. Introduction

A growing number of studies indicate that stem cells seeded on a biomaterial respond to the surrounding environment by activating nanoscale interactions with extracellular milieu following a scheme that recapitulates the tissue/organ interplays [1,2,3,4]. The first critical event occurs at the stem cell-material interface. This involves the materials’ surface characteristics and the living cells’ plasma membrane. In fact, several classes of proteins may be able to establish a bonding with the materials’ surface molecules, generating a biochemical signal cascade (mechanotransduction axis), that is transmitted to the cell nucleus, modulating the chromatin conformation and inducing a peculiar gene expression profile. As an outcome, stem cells could steer the phenotype toward a different specification lineage [5,6,7,8,9,10,11,12,13,14].

Thus, understanding the molecular events taking place at the stem cell-biomaterial interface is instrumental for generating a functional biohybrid system for applications in regenerative medicine.

Within this aim, we are studying the stem cell-biomaterial interaction in ex vivo models, consisting of poly(l-lactide) acid (PLLA) polymer-based films and stem cells.

Polylactic polymer is a bio-based polymer produced from renewable resources and currently is one of the most extensively used biodegradable aliphatic polyesters [15,16,17]. Many research groups have been studying PLLA polymers in various formulations: both PLLA and poly(d-lactide) (PLDA)enantiomers of PLA stereo-complex structures; films or fibrous forms; and in combination with other molecules to generate nanocomposite materials [5,16,17,18].

In this issue, we have produced fibrous and films of PLLA polymers and nanocomposites and have investigated their effects on several types of stem cells [5,7,15,19,20]. We have demonstrated that human bone-marrow mesenchymal stem cells (hBM-MSCs), murine-induced pluripotent stem cells (miPSCs), and murine embryonic stem cells (mESCs) responded to fibrous electrospun PLLA polymer in a similar way, maintaining shape, proliferation rate, adhesion, and stemness phenotype, as on conventional tissue culture polystyrene (TCP) cultures [7]. This contrasts with the acquisition of an osteogenic-differentiation lineage observed when the above stem cell types were seeded on nanocomposite PLLA/hydroxyapatite [7]. We have also uncovered that human umbilical cord matrix stem cells (hUCMSCs) cultured on films of PLLA lost the canonical fibroblast-like morphology typically observed on TCP, acquired three-dimensional spheroid conformations, and were steered toward an Epiblast-like phenotype [5]. Conversely, murine and human BM-MSCs cultured on the same films of PLLA polymer maintained the fibroblast-like morphology, the adhesion, the proliferation rate, and the multipotential properties as typically observed when cultured on TCP [19,20].

Together these findings suggested that the stem cells respond to the PLLA polymer differently, depending on the pristine polymer, the nanocomposite, and the chemical-physical properties of the material [5,7,15,19,20].

Starting from these results, we further investigated the potentials of PLLA polymer films on more types of adult stem cells.

In this work, we present data on the effects of PLLA polymer films on adult human adipose stem cells (hASCs). These cells were selected based on the mesenchymal origin, as hBM-MSCs and hUCMSCs previously investigated, and on their potentials in regenerative medicine application [5,21,22,23].

We cultured hASCs on PLLA polymer films and evaluated their effect on stem cells in terms of morphology, adhesion, and stem cell markers. Moreover, to establish the relevance of the stem cells-PLLA interface interaction, comparative experiments were performed with hASCs, hBM-MSCs, and hUCMSCs cultured on the PLLA polymer films modified with oxygen-plasma treatment (PLLA+O_2_).

We demonstrated that hASCs grew on PLLA as spheroids. We also showed that the oxygen-plasma treatment changed the surface hydrophilicity of PLLA and that this surface modification influenced the hASCs shape and cell adhesion. hASCs lost the capability to form spheroids and maintained their original fibroblast-like morphology, typically observed on TCP. Notably, the treatment with oxygen of PLLA had no effect on hUCMSCs and hBM-MSCs that maintained the same behavior as on PLLA.

## 2. Materials and Methods

### 2.1. Preparation and Caracterization of PLLA Polymer Films

#### 2.1.1. Processing of PLLA Films

Poly(l-lactide)acid (PLLA) biodegradable polymer, with a molecular weight (*M_n_*) of 120,000 g/mol and a polydispersity index (*M_w_*/*M_n_*) of 1.27, was supplied by Purac Biochem (Amsterdam, The Netherlands). PLLA films were prepared as previously reported by the solvent casting method, in chloroform (CHCl_3_), by using 10% *w*/*v* as polymer/solvent ratio [5,20]. PLLA was completely dissolved in CHCl_3_ by magnetic stirring for 5 h and, after the mixture was casted onto a Teflon substrate and air dried at room temperature (RT) for 24 h, stirred for a further 48 h in vacuum. Films of 60 mm in diameter and 0.2 mm in thickness were obtained.

#### 2.1.2. PLLA Oxygen-Plasma Treatment Films

The surface of PLLA films were treated by means of the radio frequency (RF) plasma method under oxygen (O_2_) flow by using a Sistec apparatus (Sistec, Binasco, Italy), with a Huttinger power supply at 13.56 MHz. The films were placed into a stainless-steel chamber, evacuated for 1 h until the pressure (P) was 9 × 10^−3^ Torr. The O_2_ flow was maintained at 60 standard cm^3^/min (sccm). The deposition conditions were: power supply: 20 W; bias voltage: 220 V; pressure: 1 × 10^−1^ Torr. Treatment time was 10 min. Process parameters were selected to obtain modulated surface features, specifically, morphology and wettability, without modifying the bulk PLLA chemical properties, according to our previous works [24].

#### 2.1.3. PLLA and PLLA+O_2_ Film Characterization

The surface microstructures were analyzed by field emission scanning electron microscope (FESEM Supra 25, Zeiss, Baden-Württemberg, Germany). A piece of PLLA film (1 cm × 1 cm) was gold coated with an Agar automatic sputter-coater and then analyzed.

Water static contact angle (WCA) measurements were used to measure the wettability of PLLA and plasma-treated PLLA films. The contact angles were assessed using the sessile drop method in air using a FTA1000 analyzer. Drops of 20 µL (high-performance liquid chromatography grade water) were placed on films and measurements were recorded 10 s after the liquid made contact with the surface.

PLLA bulk properties. Mechanical properties were performed by the tensile test method in a digital Lloyd testing machine, on rectangular samples. Infrared spectroscopy was carried out in ATR mode, by using a JASCO FT-IR 615 spectrometer (Cremella, Italy). Thermal properties were analyzed by differential scanning calorimeter (DSC, Mettler Toledo 822/e, Milano, Italia) and were conducted from −25 to 210 °C, at 10 °C min^−1^, with two heating and one cooling scans.

#### 2.1.4. Protein Adsorption

Protein adsorption assessments were performed by transferring on PLLA and on PLLA+O_2_ film surfaces 200 µL of: bovine serum albumin (BSA 2 mg/mL, Sigma Aldrich, St. Louis, MI, USA), fetal bovine serum 2% (FBS, Euroclone, Pero, Italy), FBS 10% (Euroclone), and plasma from normal donors at a dilution of 1:10 (5 mg/mL). Proteins were incubated for either 30 min or 24 h at 37 °C, according to our previous work [6]. After three washing steps in H_2_O, total protein content was measured by the Bradford method using BSA as reference curve standard. Absorbance (595 nm) was measured using a microtiter plate reader (ELISA reader, GDV-DV990BV6, Roma, Italy) [25]. Every sample was analyzed in three independent experiments. Data reported are the mean value ± the standard error of the mean of each group.

### 2.2. Isolation and Culture of Human Adult Stem Cells

#### 2.2.1. Adipose Stem Cells

Adipose stem cells were isolated from lipoaspirate adipose tissue according to our procedure [26,27]. Lipoaspirate was obtained from healthy donor patients undergoing plastic intervention, after collecting written consent, according to Ethical Committee’. Briefly, after extensively washing in phosphate-buffered saline (PBS) containing 5% penicillin/streptomycin (EuroClone, Pero, Italy), lipoaspirate fragments were incubated 40 min at 37 °C, 5% carbon dioxide (CO_2_), with 0.075% collagenase type I prepared in PBS containing 2% penicillin/streptomycin for tissue digestion, and then neutralized by adding 5 mL of DMEM (Dulbecco’s Modified Eagle Medium, EuroClone, Pero, Italy) containing 20% heat inactivated fetal bovine serum (FBS, EuroClone, Pero, Italy). The digested fragments were centrifuged at 300× *g*, the pellet washed with PBS 2% penicillin/streptomycin, and centrifuged at 300× *g* for 5 min. Finally, the cell pellet was re-suspended in growth medium (DMEM) supplemented with FBS 10%, 1% l-glutamine (EuroClone, Pero, Italy), 1% penicillin/streptomycin) plated in tissue culture flasks (TCP) and incubated at 37 °C, 5% CO_2_. hASCs started to grow as adherent fibroblast-like cells. The medium was changed every three days.

FACScan flow cytometry. To assess the cell phenotype, isolated stem cells underwent flow cytometry analysis. Stem cells were fixed on 3.7 paraformaldehyde and incubated with the following conjugated antibodies in PBS, pH 7.2, 0.5% BSA, and 2 mM EDTA: Integrin beta-1/CD29 antibody (MEM-101A), FITC conjugate; CD90/Thy-1 antibody (eBio5E10), FITC conjugate; CD105/Endoglin Antibody (SN6), RPE conjugate; CD44/H-CAM antibody (IM7), PerCP-Cy5.5 conjugate; CD45/PTPRC antibody (HI30), Pacific Orange conjugate, all from Molecular Probes. The cells were analyzed with the Attune^®^ Acoustic Focusing Cytometer (Thermo Fisher Scientific, Waltham, MA, USA) cytofluorometer. The same procedure was used to analyze the phenotype of hASCs after culture on PLLA and on PLLA+O_2_ films. Cells that acquired spheroid conformation were previously disaggregated with 0.05% Collagenase P (Roche, Basel, Switzerland), 2′ RT, and mechanical pipette action. Cells were electronically gated according to light-scattering properties to discriminate cell debris. Isotype-matched nonspecific antibodies were used as a negative control and 5000 events were recorded per each condition.

#### 2.2.2. Bone Marrow-Mesenchymal Stem Cells

hBM-MSCs were isolated from bone marrow obtained during washouts of the medullary cavities of the femurs of patients undergoing primary total hip replacement and cultured as previously described [6,7,28]. Informed consent was obtained from all donors and the institutional ethical committee approved the procedures. Mononuclear cells were isolated according to density gradient on lympholyte (Cedarlane Laboratories Limited, Hornby, ON, Canada) and were seeded in culture flasks at a density of 2.5 × 10^6^ cells in control medium consisting of RPMI-1640 (Euroclone, Milano, Italy) medium containing FBS 10%, 2 mM l-glutamine, and 1% penicillin–streptomycin (Euroclone) in a humidified atmosphere and 5% CO_2_ at 37 °C. After 5 to 7 days, the non-adherent cells were removed, and fresh medium was added to the flasks. After 15 days, a fibroblast-like colony started to grow. The medium was changed every three days.

#### 2.2.3. Umbilical Blood Matrix Stem Cells

hUCMSCs were isolated from matrix of cord blood and expanded in culture according to our protocol [5,29]. At the end of gestation, human umbilical cords, collected after caesarean deliveries, under official consent of the Hospital Board at University of Perugia (Perugia, Italy) and patient’s own informed consent was sent us from the Department of Obstetrics and Gynecology. The cord was cut into pieces (about 10 cm) and injected by a syringe with the digestion solution: 77 mM NaCl, 0.1 mg/mL bovine serum albumin (BSA, Biochrom, Biopsa, Milan, Italy), 1.5 mg/mL hyaluronidase (Sigma Aldrich), 0.5 mg/mL liberase purified enzyme blend (Roche, Milan, Italy), in 0.02 M phosphate buffer at pH 7, containing 77 mM NaCl and 0.01% BSA, pre-warmed at 37 °C. The tissue digest was first spun at 1500 rpm for 5 min at 4 °C, and thereby resuspended in DMEM with antibiotics. Residual RBC’s were removed by Lymphoprep^TM^ gradients at 1850 rpm for 20 min at 4 °C. hUCMSCs were seeded at a concentration of 250,000 per flask pre-treated with hyaluronic acid, in FBS 2% culture medium, according to Weiss [30]. After 24 h, EGF (1 ng/mL) (Peprotech, LiStarFish, Milano, Italy) and PDGF-BB (10 ng/mL) (Peprotech) were added to the culture flasks. The cells were maintained at 37 °C in a humidified atmosphere with 5% CO_2_. Cell expansion throughout 80% confluence was achieved by treatment with 0.05% trypsin/EDTA (Gibco, Invitrogen, Milan, Italy) for 3 min at 37 °C. The medium was changed every three days.

### 2.3. Culture of Human Adipose Stem Cells on PLLA and PLLA+O_2_ Films

PLLA and PLLA+O_2_ films were cut into 1 cm^2^ snippets squares, sterilized through immersion in pure ethanol for 30 min, followed by five rinses in PBS, and then deposited in a 24-well plate. Stem cell suspensions of 3 × 10^3^ stem cells were seeded dropwise, on sterilized films, and 500 μL of culture medium was gradually added to each snippet. Stem cell-PLLA and -PLLA+O_2_ cultures were incubated at 37 °C in a humidified atmosphere with 5% CO_2_. The medium was changed every three days. As an internal control, experiments were performed seeding stem cells on tissue culture polystyrene (TCP). Cultures were conducted at different time points (from day 0 to day 21) and evaluated for proliferation, viability, morphology, and expression of stem cell markers.

### 2.4. Culture of Human BM-MSCs and Human UCMSCs on PLLA and PLLA+O_2_ Films 

Stem cell suspensions of 3 × 10^3^ stem cells (both hBM-MSCs and hUCMSCs) were seeded dropwise on 1 cm^2^ snippet squares of PLLA and PLLA+O_2_ films, as above described. Stem cell-PLLA and -PLLA+O_2_ cultures were incubated at 37 °C in a humidified atmosphere with 5% CO_2_. The medium was changed every three days. As an internal control, experiments were performed by seeding stem cells on TCP.

### 2.5. Cells Viability Assay

Stem cell viability was evaluated by incubating 3 × 10^3^ cells/mL cultured on PLLA and PLLA+O_2_ at different time points (1, 2, 3, 5, 7, 14, and 21 days) with XTT (2,3-bis[2-Methoxy-4-nitro-5-sulfophenyl]-2H-tetrazolium-5-carboxyanilide inner salt) (Sigma Aldrich) according to the manufacturer’s recommendation and our previous works [5,26]. As an internal control, parallel experiments were conducted by seeding stem cells on TCP. Interference effects of PLLA and PLLA+O_2_ snippet squares without cells on XTT assay were also considered.

The absorbance of the samples was measured using a microtiter plate reader (ELISA reader, GDV, Roma, Italy) at 450 nm with a reference wavelength at 650 nm.

The presence of dead cells was monitored by counting stem cells in a haemocytometer by using the Trypan Blue reagent.

### 2.6. Immunofluorescences 

Immunostaining was performed as previously described [5,7,28]. Briefly, cells on PLLA and on PLLA+O_2_ snippet squares were rinsed twice with PBS, fixed in 4% paraformaldehyde for 20 min and, after PBS washing, permeabilized and incubated in blocking solution (PBS + FBS 10%, 0.1% Triton X-100) for 1 h at room temperature (RT). Samples were incubated with phalloidin (Alexa-fluor-488 phalloidin, Invitrogen, Grand Island, NY, USA) for 20 min, or overnight at 4 °C with several primary antibodies: anti-Vinculin (Abcam, Cambridge, UK), anti-Myosin-IIA (Santa Cruz Biotechnology, Inc., Dallas, TX, USA), anti-Filamin-A (Santa Cruz Biotechnology, Inc., Dallas, TX, USA), and anti-Lamin-B (Santa Cruz Biotechnology, Inc., Dallas, TX, USA). In the latter cases, after being washed with PBS, they were stained with Alexa-Fluor 488-nm conjugated secondary antibodies (Invitrogen) for 1 h at room temperature.

Finally, after being washed with PBS, samples were mounted and nuclei were counterstained with Vectashield^®^ with DAPI (4,6-diamidino-2-phenylindole; Vector Laboratories Inc., Burlingame, CA, USA).

Immunofluoresces were evaluated by fluorescence microscope Eclipse-TE2000-S (Nikon, Tokyo, Japan) equipped with the F-ViewII FireWire camera (Soft Imaging System, Olympus, Germany).

Interference of PLLA and PLLA+O_2_ snippet squares without cells was evaluated to the fluorescence microscope.

### 2.7. Image Analysis

The nuclear shape index [5] was evaluated in cells cultured on each substrate. Five different areas were photographed (20× magnification, 20× Plan Fluo NA0.5) and an average of 300 nuclei were analyzed. To quantify the variation of nuclear shape index (NSI), the area and perimeter of nuclei were measured by Fiji (Fiji Life-Line version, 22 December 2015) on fluorescent-stained DAPI images and used to calculate the NSI from the relationship:

(1) $$NSI=\frac{\left(4\pi \;\times \;\mathrm{area}\right)}{{\mathrm{perimeter}}^{2}}$$

*NSI* values range from 0 (elongated, elliptic morphology) to 1 (circular shape).

The edge detection method was used to detect the location of the leading edge with a custom-written automated method in MATLAB software that automatically generate the Prewitt image.

Briefly, the image was imported in ‘*RGB.bmp*’ format *(imread)* and doubled *(im2double)*. The Prewitt operator was applied to the doubled image by specifying a 5 × 5 mask, with the two convolution kernels *Gx* = [2 2 4 2 2; 1 1 2 1 1; 0 0 0 0 0; −1 −1 −2 −1 −1; −2 −2 −4 −2 −2] and *Gy* = [2 1 0 −1 −2; 2 1 0 −1 −2;4 2 0 −2 −4; 2 1 0 −1 −2; 2 1 0 −1 −2], which are convolved with the original image to calculate approximations of the derivatives (one for horizontal and one for vertical changes). The final image G=$\sqrt{Gx^{2}+Gy^{2}}$ for each RGB channel was saved.

### 2.8. Field Emission Scanning Electron Microscopy (FESEM)

Stem cell–film interaction was evaluated by FESEM at each time point of culture. Samples were rinsed twice with PBS and fixed in 2.5% glutaraldehyde for 30 min at RT, then dehydrated by adding progressively more concentrated ethanol (5–100% *v*/*v*) every 5 min, and finally dried by the critical point machine (CPD, Emitech K850). Once dried, the samples were gold sputter-coated before examination by FESEM (Supra 25 Zeiss), at an accelerating voltage of 5 kV.

### 2.9. Statistical Analysis

Data analyses were reported as the mean ± SEM (GraphPad 4.03 Software, San Diego, CA, USA, 2011). *p* ≤ 0.05 was considered statistically significant.

## 3. Results

### 3.1. PLLA and PLLA+O_2_ Film Characterization 

**Figure 1:**
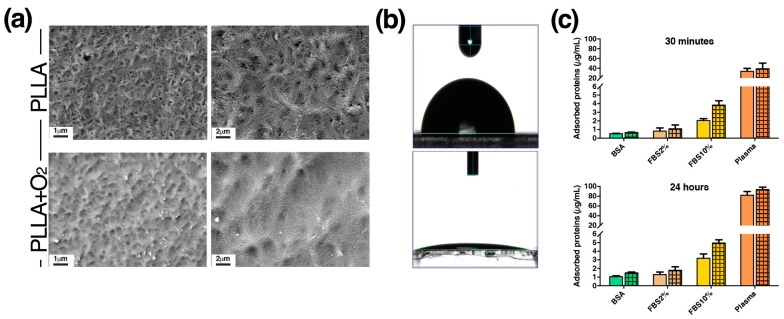
PLLA and PLLA+O_2_ polymer films. (**a**) Representative FESEM images of PLLA and PLLA+O_2_ surface morphology; (**b**) WCA images of PLLA and PLLA+O_2_ surfaces (see method for details); and (**c**) protein adsorption is reported as mean ± SEM. Clear fill bar: PLLA; patterned fill bar: PLLA+O_2_.

In this work we developed films of pristine poly(l-lactide)acid (PLLA) polymer and films of PLLA polymer treated with oxygen-plasma (PLLA+O_2_) (Figure 1). Films of PLLA were made according to our previous works by using the solvent casting procedure [5,20]. PLLA polymer films were optically transparent and have a thickness of 250 μm. Mechanical property measurements showed a Young’s modulus of 680 MPa and an elongation at break of 144%. No variations were observed in oxygen treated samples.

DSC analysis revealed that the plasma surface treatment did not affect the thermal properties of the PLLA polymer film. The melting temperature was about 172 °C for neat PLLA and for PLLA oxygen treated samples, with a percentage of crystallinity around 50% for all systems. Thus, the oxygen plasma process did not affect the characteristic thermal transitions of the polymer matrix.

The overall data confirmed our previous findings [24,31] and indicated that the treatment with oxygen-plasma modify the surface of the pristine PLLA without changing PLLA’s bulk properties.

#### 3.1.1. Surface Morphology Characterization of PLLA and PLLA+O_2_

FESEM images show the surface morphology of PLLA and PLLA+O_2_ films. Pristine PLLA presented a detectable roughness with a specific topology (e.g., parallel lines) that was induced by the Teflon topography, used for the casting samples’ preparation (Figure 1a). Images also revealed the etching effect of the oxygen plasma treatment of PLLA (Figure 1a). It is well known that the plasma treatment of surfaces gave a mass reduction due to ion etching [31]. Increasing the magnification, the images show the presence of nanostructured surfaces, induced by the oxygen treatment.

#### 3.1.2. Wettability Measurements

Water static contact angle (WCA) measurements were performed to study the effects of plasma treatment on surface wettability. Due to PLLA’s hydrophobic nature, PLLA films have hydrophobic behaviors. Ten minutes of radio frequency oxygen-plasma treatment with RF fixed at 30 W of power decreased the water contact angle of the pristine PLLA from 90° to less than 10°, changing the surface’s properties from hydrophobic to hydrophilic (Figure 1b).

Modulating the parameters of the oxygen treatment we may control the wettability properties. Low power supply values (i.e., 10 W, [24]) permit increasing the wettability, obtaining materials with 50° WCA. Hence, the power level and treatment time were selected in order to obtain lower values of WCA and, at the same time, to maintain the polymer’s chemical stability. According to our study [24], spectra analysis confirmed that this treatment did not induce chemical changes in the carbonyl (1756 cm^−1^) and in the C–O–C (1080 cm^−1^) stretching peaks (data not shown).

#### 3.1.3. Protein Adsorption

Figure 1c shows the adsorption of BSA (2 mg/mL), FBS 2%, FBS 10%, and plasma (5 mg/mL) on PLLA and on PLLA+O_2_ films at two different time points, 30 min and 24 h, of protein incubation.

We observed an increase of adsorption level of FBS 2%, FBS 10%, BSA, and plasma on PLLA+O_2_ compared to PLLA. The increase was higher at 24 h compared to 30 min of incubation on both polymer films and was more evident for FBS 10% and plasma (Figure 1c). The highest increase in protein adsorption on both films of plasma and FBS 10% (>FBS 2%) is a consequence of the highest protein concentration in these samples.

### 3.2. Culture of hASCs on PLLA and PLLA+O_2_

**Figure 2:**
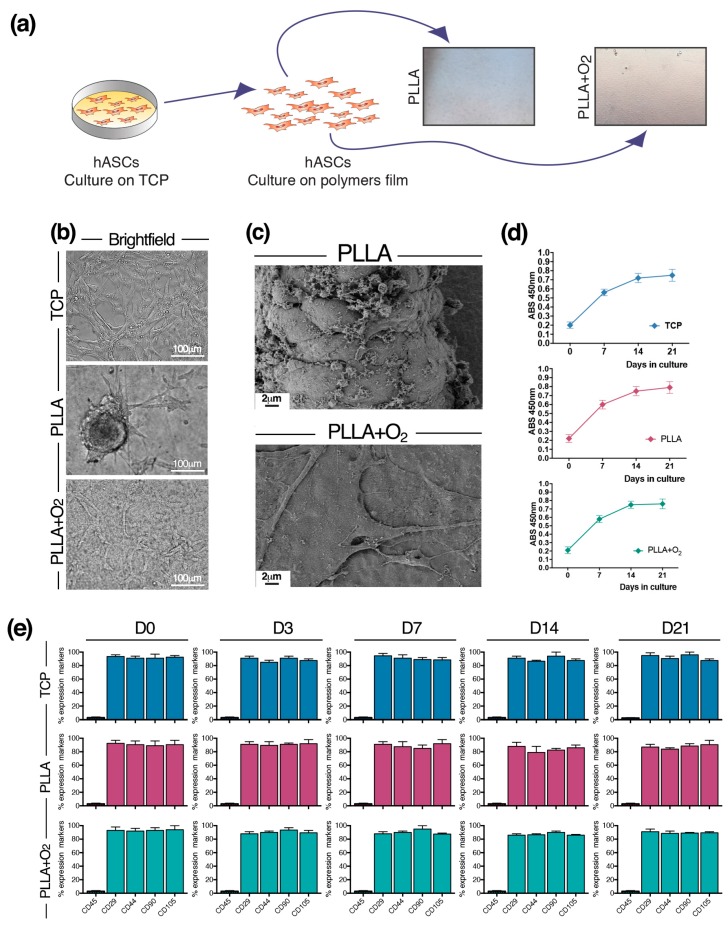
hASCs cultured on PLLA and PLLA+O_2_ polymer films. (**a**) Schematic of hASCs on PLLA and PLLA+O_2_ culture platforms; (**b**) brightfield representative images of hASCs on TCP, PLLA, and PLLA+O_2_; (**c**) FESEM representative images highlight the hASCs spheroids organization on PLLA film and the monolayer fibroblast-like shape on PLLA+O_2_ film; (**d**) hASCs viability (XTT assay) on TCP, on PLLA, and on PLLA+O_2_; and (**e**) expression of mesenchymal stem cell markers in hASCs at different time points of culture (0, 3, 7, 14, 21) on TCP, on PLLA, and on PLLA+O_2_. Average values for mesenchymal phenotype markers shown by hASC (*n* = 3) are reported as the mean ± SEM.

hASCs were seeded on films snipped into squares (Figure 2a). hASCs grew generating spheroids attached to the PLLA surface. On the contrary, hASCs cultured on PLLA+O_2_ films maintained the canonical monolayer fibroblast-like morphology, typically observed on TCP (Figure 2b).

The different cellular organization of hASCs on PLLA and on PLLA+O_2_ films is also revealed by FESEM images. While on films of PLLA hASCs were strictly packed together in a spheroid conformation, on PLLA+O_2_ films the stem cells grew adherent to the polymer surface in a monolayer organization (Figure 2c).

hASCs viability was not affected by the characteristics of both PLLA films. We observed a similar dehydrogenase activity in spheroids hASCs on PLLA, in fibroblast-like hASCs on PLLA+O_2_, and on TCP (Figure 2d). No dead cells were observed during in vitro cultures’ maintenance, as confirmed by Trypan Blue assay.

We investigated the expression of stem cell markers in hASCs cultured on PLLA and on PLLA+O_2_. We found a similar expression level of mesenchymal stem markers in hASCs cultured on TCP, on PLLA, and on PLLA+O_2_ (Figure 2e). The levels of CD29, CD44, CD90, and CD105 markers were comparable in stem cells at the time point of seeding on each substrate (day 0) and remained stable over the time in culture (days 3, 7, 14, and 21) (Figure 2e). No expression of the CD45 marker was detected in all samples at each time point (Figure 2e), confirming the non-hematopoietic origin of hASCs [21,22]. These results indicate that the oxygen treatment did not influence the stemness property of hASCs.

### 3.3. Morphology of hASCs on PLLA and PLLA+O_2_

Stem cell shape was revealed by cytoskeleton F-actin staining. The analysis was performed at days 3, 7, 14, and 21. Reported images are representative of these time points.

**Figure 3:**
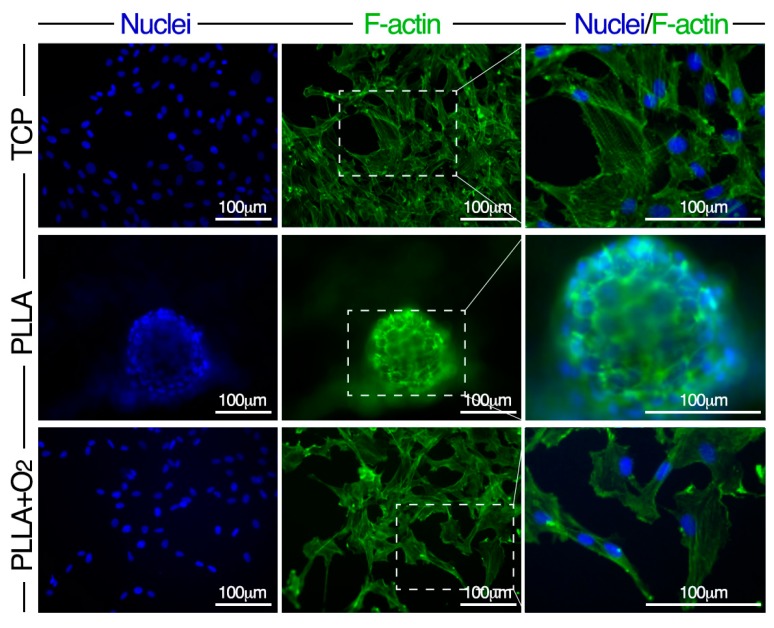
Morphology of hASCs on PLLA and on PLLA+O_2_ cultures. Representative fluorescence images of nuclei (DAPI, blue) and F-actin (Phalloidin-FITC, green) in hASCs on TCP, on PLLA, and on PLLA+O_2_. High magnification of selected dashed squares (merged DAPI-Phalloidin) is reported in the right panel.

In hASCs on TCP and on PLLA+O_2_ films F-actin stress fibers crossed the cells traversing the cytoplasm and were almost oriented parallel to the main cellular longitudinal axis (Figure 3). Conversely, in hASCs on PLLA polymer films, F-actin fibers showed a circular organization, following the perimeter of the cells that had changed shape from elliptical to rounded (Figure 3).

We also monitored the expression of the cytoskeleton actin-linking proteins Filamin-A and Myosin-IIA (MHIIA). These proteins specifically interact with F-actin fibers to generate a three-dimensional structure determining cells’ shape [32,33]. Moreover, together with the F-actin, Filamin-A and Myosin-IIA are the targets of cues generated by the adhesion complexes [32,33] and, therefore, are informative on the interaction between polymers and cells.

**Figure 4:**
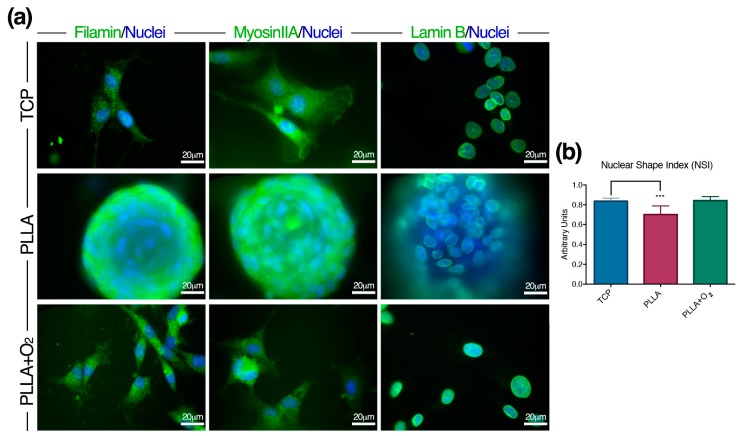
hASCs architecture on PLLA and on PLLA+O_2_. (**a**) Representative immunofluorescence images of Filamin-A, Myosin-IIA and Lamin-B proteins in hASC spheroids on PLLA, and fibroblast-like hASCs on PLLA+O_2_ and on TCP; and (**b**) NSI in hASCs on PLLA, PLLA+O_2_, and TCP (see methods for details). *** *p* ≤ 0.001.

We found different expression of Filamin-A and Myosin-IIA proteins between spheroids and fibroblast-like monolayer hASCs (Figure 4a). In hASCs organized in spheroids on PLLA, both proteins depicted a circular organization of the cytoskeleton; on the contrary, they organized as elliptic structures in fibroblast-like hASCs on PLLA+O_2_ (Figure 4a).

Finally, we monitored the expression of Lamin-B, one of the nucleoskeleton proteins connecting the cytoskeleton proteins and the nucleus [34,35]. Lamin-B expression followed the change of the nuclear shape as a consequence of the spheroid organization on pristine PLLA. On the contrary, Lamin-B designed a canonical nuclear shape in ASCs on PLLA+O_2_ and on TCP (Figure 4a).

We also measured the NSI in stem cells cultured on pristine PLLA and on oxygen-plasma treated PLLA polymer films (Figure 4b). We observed a reduction of NSI in hASCs organized in spheroids on PLLA. This finding indicated a nuclear stretching, possibly influencing chromatin conformation. No variations of NSI in hASCs growing on PLLA+O_2_ or on TCP were detected (Figure 4b).

### 3.4. Adhesion of hASCs on PLLA and on PLLA+O_2_ Surfaces

**Figure 5:**
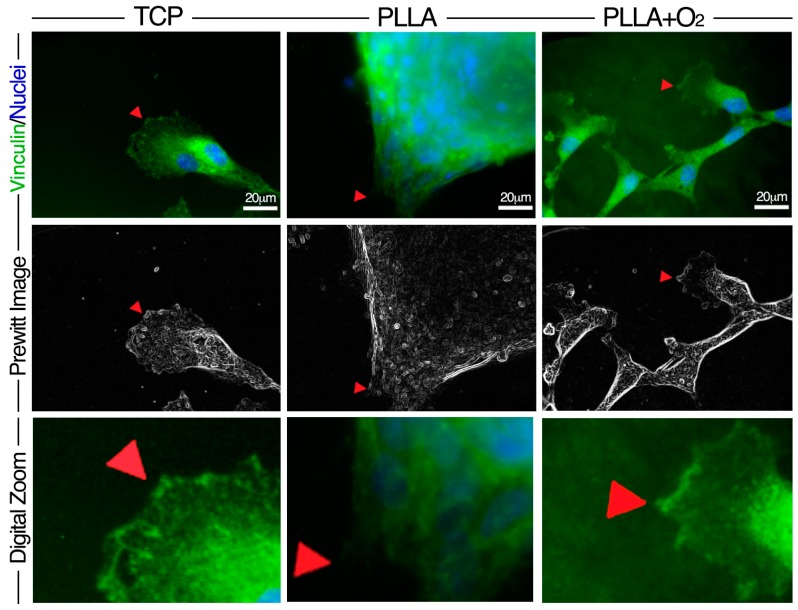
Distribution of Vinculin Focal Adhesion Spots in hASCs on PLLA and on PLLA+O_2_. Immunofluorescence representative images show the expression of Vinculin (green) and VFASs (red arrow) and the related Prewitt images elaboration by MATLAB software. Image magnification: 60×. Digital zoom 3× from the original 60× images.

We studied the adhesion of hASCs on PLLA and on PLLA+O_2_ surfaces analyzing the expression of the focal adhesion protein Vinculin, classically involved on the cellular-matrix interaction [5,36,37]. The analysis was performed at days 3, 7, 14, and 21. Reported images are representative of these time points (Figure 5).

Within spheroids’ conformation, Vinculin focal adhesion spots (VFASs) converged in small areas allowing the robust adhesion of hASCs to the surface of PLLA polymer films. Oppositely, monolayer fibroblast-like hASCs engaged VFASs in large areas on the surface of PLLA+O_2_ polymer films as on TCP (Figure 5). The VFASs organization at the hASC-polymer film interface is highlighted by the corresponding Prewitt images obtained by MATLAB software. Images revealed a fan-shaped contact area distribution of VFASs in stem cells on PLLA+O_2_ films and on TCP while they showed an organization in a small convergent contact area in hASCs on PLLA polymer films (Figure 5).

### 3.5. hASCs Spheroids Formation on PLLA

**Figure 6:**
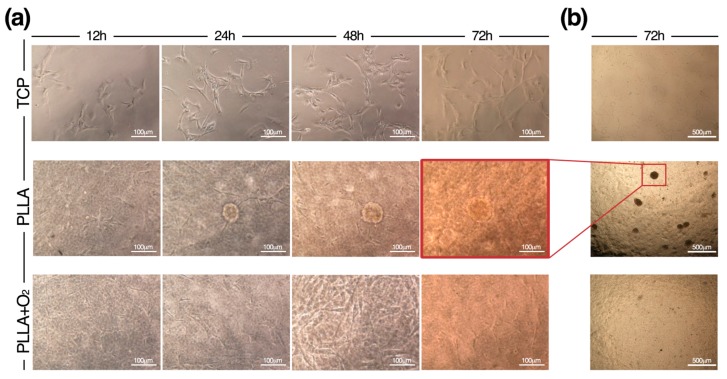
Time-to-time formation of hASC spheroids on PLLA. (**a**) Representative brightfield images by an Eclipse-TS100 microscope equipped with a Nikon camera (Nikon, Tokyo, Japan), show the spheroids’ organization of hASCs during the culture period on PLLA films and their canonical fibroblasts-like morphology on PLLA+O_2_ film and on TCP; and (**b**) representative overview of hASCs on TCP, on PLLA, and on PLLA+O_2_ films.

In Figure 6 are reported the time-to-time formation of hASC spheroids on PLLA polymer films. hASCs acquired spheroids conformation rapidly, indicating that stem cells responded to the surface characteristic of PLLA immediately after cell seeding. Spheroids appeared after 24 h and their number and dimension increased during culture maintenance, suggesting that spheroids are consequence of cell aggregation (Figure 6). This contrasted with the culture of hASCs on PLLA+O_2_ where stem cells retained the native morphology as on TCP (Figure 6).

### 3.6. Effect of PLLA Oxygen-Plasma Treatment on hBM-MSCs and hUCMSCs Cultures

In order to establish whether the oxygen-plasma treatment to PLLA influnces the adhesion of other adult human stem cell types we perfomed comparative experiments using hBM-MSCs and hUCMSCs. These stem cells were selected based on our previuos studies exploring their interaction with the surface of PLLA polymer films [5,20], as well as on their mesenchymal origin [21,22,23].

**Figure 7:**
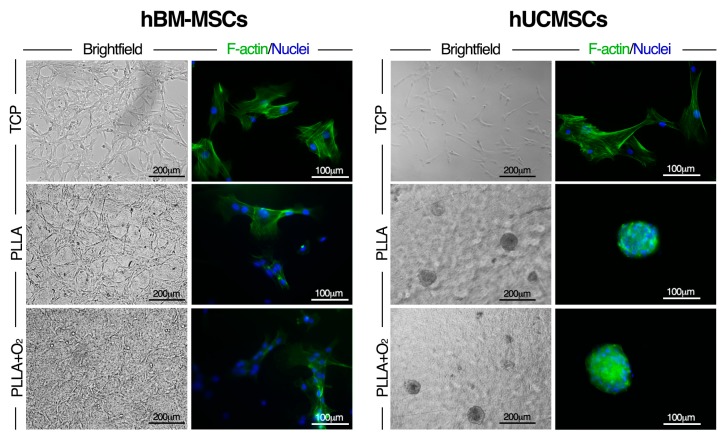
Effect of oxygen-plasma treatment to PLLA on hBM-MSCs and hUCMSCs cultures. Representative brightfield and immunofluorescence images of hBM-MSCs and hUCMSCs cultured on TCP, PLLA, and PLLA+O_2_, respectively. F-actin (Phalloidin-FITC, green) and nuclei (DAPI, blue).

The oxygen treatment of PLLA did not change hBM-MSCs performances. hBM-MSCs maintained the same fibroblast-like shape and viability behaviours on PLLA and on PLLA+O_2_ as on TCP (Figure 7). Maintainace of the canonical mesenchymal shape by hBM-MSCs on both PLLA films as typically observed on TCP was confirmed at molecular level by F-actin immunostaining (Figure 7).

No changes in shape were observed when hUCMSCs were cultured on PLLA+O_2_ polymer films. In fact, immufluorescences staining and brightfield inspection revealed that the spheroid conformation, typically generated by hUCMSCs on PLLA polymer films [5], was also acquired by hUCMSCs cultured on PLLA+O_2_ (Figure 7). hUCMSCs cultured on TCP as reference, mainatined their fibroblast-like morphology (Figure 7).

These results suggest that the wettability properties of PLLA surfacs are not involved in the formation of hUCMSC spheroids nor in the hBM-MSCs adhesion.

## 4. Discussion

In this work, we demonstrated that PLLA polymer films’ surface properties guide hASCs toward a multicellular three-dimensional spheroid conformation instead of a monolayer fibroblast-like morphology as typically observed on TCP. Noteworthy, we demonstrated that the PLLA film surface hydrophilicity is involved in the acquisition of a spheroid conformation by hASCs, since the treatment of the PLLA film surface with plasma-oxygen maintained the fibroblast-like morphology of hASCs. Interestingly, the oxygen treatment had no effect on the performance of hBM-MSCs and hUCMSCs cultured on PLLA+O_2_ as they maintained the same cell shape observed on PLLA polymer film.

Modification of the PLLA surface is commonly used to modulate the affinity of this polymer with the cells [38,39,40,41,42]. Many different functional groups, including diethylenetriamine, 2-(2-aminoethoxy)ethanol or GRGDS (cell adhesion peptide) [40], aldehyde and amine groups [41], and poly(sulfobetaine methacrylate)-catechol conjugates [42] have been used to improve stem cells adhesion and proliferation on PLLA polymer (both fibrous and film forms).

The plasma oxygen treatment is a useful method to spread hydroxyl groups on the surface of several polymers [43], including PLLA [44,45,46,47], in order to improve cellular adhesion to their surface.

In our study, after oxygen-plasma treatment, the surface of PLLA films (PLLA+O_2_) became hydrophilic and its surface nanoroughness increased, whereas the hydrolytic degradation properties of PLLA were not affected [31]. In agreement with other studies [24,31,44,45,46] we demonstrated the increase of BSA, FBS 2%, FBS 10%, and plasma adsorption on the hydrophilic PLLA+O_2_ surface compared to protein adsorption on the hydrophobic surface of PLLA polymer films. Moreover, according to our previous work, RF oxygen-plasma surface modification did not affect PLLA bulk properties [24,31]. These data confirmed our previous evidence on the adsorption of FITC-BSA, showing how plasma treatment modulates the PLLA surface properties and induces the formation of the surface nanostructure of different shapes affecting the hydrophilic/hydrophobic behaviors without changing the PLLA bulk properties [24,31]. Furthermore, in vitro degradation studies performed in physiological conditions have showed that plasma treatment does not affect the PLLA bulk and the hydrolytic degradation properties [31].

Thus, oxygen treatment allows the production of two distinct PLLA polymer films, differing for hydrophilicity and WCA (PLLA > PLLA+O_2_), representing a suitable model for studying the effect of the polymer film on stem cells.

Compared to the in vitro culture on TCP, where hASCs adhere and display a monolayer fibroblast-like shape, adipose stem cells cultured on PLLA grew generating three-dimensional multicellular organizations (spheroids), strongly attached to the film’s surface. We suggest that this new cellular organization is the consequence of the adipose stem cell-material interaction. Several studies have suggested that stem cells may change their shape depending on signals they receive from external milieu through the focal adhesion (FA) proteins, directly involved in tethering with the extracellular matrix [10,11,12,13,48,49]. In this contest, Vinculin plays a central role in organizing the FA complexes (VFASs) [36] and contributes to the organization of the cytoskeleton architecture [37]. In our system, the different VFASs’ distribution of hASCs on PLLA (fan-shaped) and on PLLA+O_2_ (convergent area) significantly influenced the cytoskeleton F-actin organization, along with Filamin-A and Myosin-IIA (two F-actin-linking proteins involved on the control of the cell stiffening [32,33] and, therefore, in stem cells morphology acquisition). It is likely that the difference in hydrophilicity between the two surfaces interferes with hASCs’ adhesion to the polymer films surface and drives the stem cells to acquire different conformation: spheroids on PLLA and monolayer fibroblasts-like on PLLA+O_2_.

According with other reports [43,44,47], our results confirmed that the oxygen-plasma treatment improves the adhesion and spreading of hASCs on the polymer films. Nevertheless, surface modification neither changed the stem cells’ proliferation rate, nor stemness markers’ expression compared to hASCs at the time of seeding (day 0). Levels of mesenchymal stem cell markers were comparable in hASCs seeded on PLLA and on PLLA+O_2_ during the culture period (from day 0 to day 21) and were similar to those measured in hASCs cultured on TCP [26,27]. These results are in agreement with our previous finding, showing that the PLLA polymers maintain the stem cells’ properties [5,7,19,20].

Of note, our work adds new finding on the effects of the hydrophilicity on the final cellular organization and points out the role of the stem cell’s type with respect to this phenomenon.

The results that we obtained by culturing adult human UCMSCs and BM-MSCs on PLLA and PLLA+O_2_ polymer films may be explained in this context. In fact, after seeding on PLLA+O_2_, hUCMSCs maintained the spheroid conformation they had on PLLA polymer film, however, different from the fibroblast-like monolayer morphology typically observed on TCP [5]. No variations in cellular organization were found in hBM-MSCs cultured on PLLA+O_2._ These cells maintained the canonical monolayer fibroblast-like shape as on PLLA film and on TCP [20]. Together, these findings indicate that PLLA+O_2_ surface hydrophilicity is not directly involved in driving the architecture of hBM-MSCs and the hUCMSCs on the polymer films. We may explain the formation of spheroids by hASCs and hUCMSCs on PLLA polymer films and, foremost, the absence of hASC spheroids rather than the maintenance of hUCMSC spheroids on PLLA+O_2_ polymer films, as the consequence of specific molecular differences at the stem cell interface with the film. More experiments are needed to identify the origin of this phenomenon. However, according to other studies [5,8,9,10,11,12], these findings support the hypothesis that, at the cell-material interface, stem cells and the material assist each other. This causes an active cross-talk linking the molecular cellular mechanisms with the intrinsic materials’ properties to generate a selected cell response (e.g., stem cells shape, adhesion, or functions).

Collectively, our results highlight the role of surface modifications as suitable tools to study stem cells-material interfaces. Moreover, as the interaction might be stem cells specific, they point out the need to perform such experiments considering more types of stem cells.
